# Severe COVID-19 as a Possible Mediator of Autoimmunity and Sjögren’s Syndrome

**DOI:** 10.7759/cureus.35290

**Published:** 2023-02-22

**Authors:** Kazuhisa Konishi, Hiroomi Kuwahara, Yasuko Fujimoto, Kazuhiro Nagata, Jun Takeda

**Affiliations:** 1 Department of Respiratory Medicine, Koseikai Takeda Hospital, Kyoto, JPN; 2 Department of Otolaryngology, Kyoto Teishin Hospital, Kyoto, JPN; 3 Department of Diabetes and Endocrinology, Koseikai Takeda Hospital, Kyoto, JPN

**Keywords:** covid-19 respiratory failure, sjögren’s syndrome, long-haul covid, autoimmnity, covid-19

## Abstract

We treated a 65-year-old man for COVID-19 who was hospitalized urgently due to life-threatening respiratory decompensation and later developed cardiac arrest, both of which were successfully treated. Three days prior to the patient's urgent hospitalization, he had a high fever of over 38.0°C. Viral infection was diagnosed by polymerase chain reaction (PCR) on the day of admission, which was negative on the 11th day. Blood analysis on the second day was strongly positive for COVID-19 IgG antibodies, which continued for one year. Because of the acute increase in viral IgG antibodies, we performed other immunological analyses; Sjögren's syndrome antigen A (SS-A) and Sjögren's syndrome antigen B (SS-B) antibodies were positive, although he had no history of autoimmune diseases. Subsequent salivary-gland biopsy and pathological analysis confirmed the diagnosis of Sjögren’s syndrome. The severe clinical manifestations and early antibody seroconversion in this case suggest COVID-19 as a mediator of autoimmunity and Sjögren’s syndrome.

## Introduction

Severe cases of COVID-19 infection have a high rate of mortality, especially in patients requiring treatment in the intensive care unit [1]. A growing number of post-COVID-19 manifestations appearing after eradication of the viral infection have been reported, and these differ among patient populations [2,3]. Increasing observations and other evidence suggest that autoimmunity is involved in these clinical and molecular features [4]. Here, we describe a patient with severe COVID-19 and early seroconversion of viral antibodies, from whom we obtained a histological diagnosis of Sjögren’s syndrome.

## Case presentation

A 65-year-old man with a pre-existing diagnosis of hypertension presented with fever, cough, and dyspnea. Three days before his initial visit, he developed a high fever of over 38.0°C, followed by other symptoms, including severe cough and dyspnea, that prompted his admission to our emergency unit via ambulance. 

**Figure 1:**
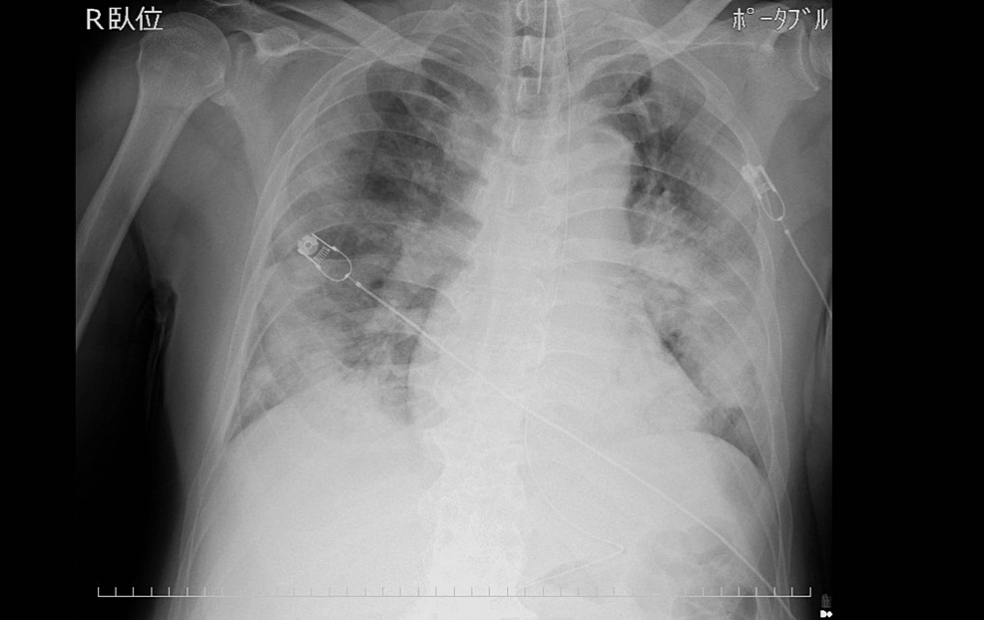
Chest x-ray at the time of admission. The chest x-ray reveals consolidation that is distributed bilaterally, reflecting severe respiratory decompensation due to COVID-19.

The patient's initial physical examination revealed a body temperature of 36.7°C, a blood pressure of 138/71 mmHg, an irregular pulse of 91 beats per minute, and a respiratory rate of 20 beats per minute. His pulse oximetry demonstrated oxygen saturation of 79% in room air, and his respiratory and circulation status deteriorated rapidly in the emergency unit. His general status included obesity (BMI: 31 kg/m^2^), a body weight of 98.3 kg, and a body height of 178 cm. COVID-19 viral polymerase chain reaction (PCR) test revealed positivity in the nasal swab sample obtained upon admission, and the chest x-ray demonstrated diffuse bilateral opacities enhanced in the subpleural areas (Figure 1). The patient had not been vaccinated for COVID-19 before the emergency visit.

Laboratory findings of his peripheral blood at the initial visit are shown in Tables 1, 2.

**Table 1:** Peripheral blood findings at the time of admission.

Peripheral blood findings	
White blood cells	8500/uL
Red blood cells	422/uL
Hemoglobin	12.5 g/dL
Hematocrit	38.1%
Platelet	17.9 /uL
Mean corpuscular volume	90 fl
Mean corpuscular hemoglobin	29.6 pg
Mean corpuscular hemoglobin concentration	32.8%
Red blood cell distribution width	13.3%
Mean platelet volume	10.5 fl
Basophils	0.2%
Eosinophils	0%
Neutrophils	80.70%
Lymphocytes	14.20%
Monocytes	4.90%
Aspartate aminotransferase	82 U/L
Alanine transaminase	94 U/L
Alkaline phosphatase	161 U/L
Lactate dehydrogenase	501 U/L
Glucose	131 mg/dL
HbA1c	6.50%
Blood urea nitrogen	19 mg/dL
Creatinine	0.93 mg/dL
Sodium	137 mEq/L
Potassium	4 mEq/L
Chloride	100 mEq/L
Calcium	7.6 mg/dL
Troponin-I	0.14 ng/mL
C-reactive protein	10.44 mg/dL
Prothrombin time	10.7 s
Prothrombin time%	92.6
Prothrombin time (international normalized ratio)	1.04
Activated partial thromboplastin time	34.7 s
Fibrinogen	603 mg/dL
Brain natriuretic peptide	75.1 pg/mL

**Table 2:** Blood gas analysis at the time of admission. Mechanical ventilation volume control (VC) mode; tidal volume 500 mL, positive end-expiratory pressure (PEEP) 7 cm H_2_O, FiO_2_ 100%, and respiratory rate 10/min.

Arterial blood gas	
pH	7.412
Partial pressure of carbon dioxide	36.7 torr
Partial pressure of oxygen	73.6 torr
Bicarbonate	22.9 mmol/L
Base excess	−1.7 mmol/L
Anion gap	11.1 mmol/L
Oxygen saturation	94.7%

**Figure 2:**
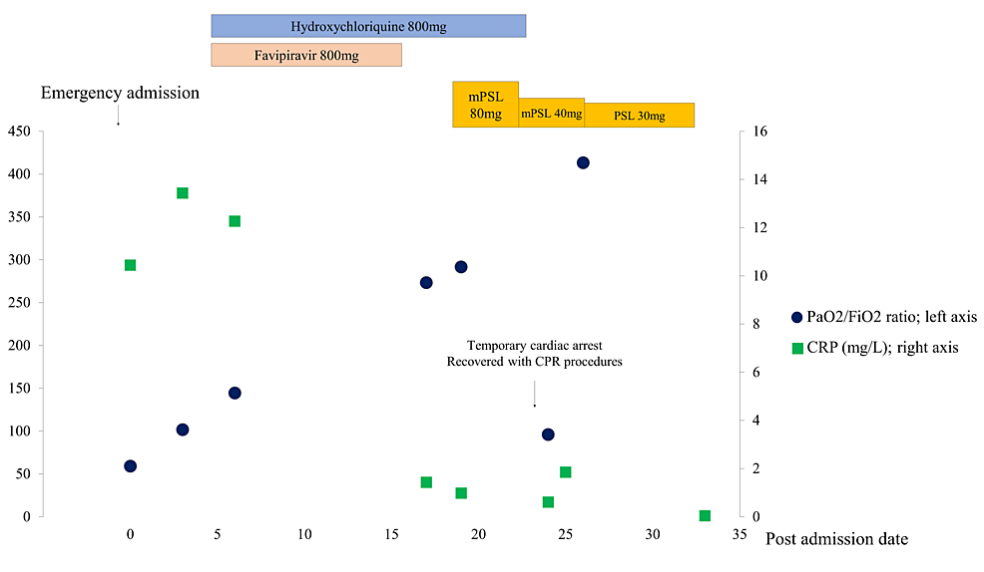
Clinical course in the ICU. The patient was administered hydroxychloroquine and favipiravir during the acute phase. Steroids were given to the patient, starting with 80 mg of methylprednisolone for three days, followed by gradual tapering (40 mg of prednisolone for four days, 30 mg of prednisolone for five days). The patient’s PaO_2_/FiO_2_ ratio values are indicated with blue circles, and serum C-reactive protein (CRP) levels are indicated with green squares. On the 24th day post-admission, a transient worsening of the PaO_2_/FiO_2_ ratio was observed, reflecting temporary cardiac and pulmonary arrest. ICU: intensive care unit.

The patient was intubated, admitted to our isolation unit, and provided respiratory support through positive pressure mechanical ventilation initially set in volume control mode with a tidal volume of 500 mL, a positive end-expiratory pressure (PEEP) of 7 cm H_2_O, a respiratory rate of 10/min, and a FiO_2_ of 100%. In addition, the airway pressure was maintained at approximately 20 cm H_2_O. Because the patient started to develop signs of circulatory failure with a drop in blood pressure to 91/59 mmHg after intubation, a higher PEEP pressure was not applied. He was also administered fluids and norepinephrine together with empiric therapy, including hydroxychloroquine [5], favipiravir [6], and broad-spectrum antibiotics. Subsequently, he received systemic steroids, initiated with 80 mg of methylprednisolone followed by a prednisolone taper, as suggested by the Infectious Diseases Society of America guidelines published when the patient was receiving treatment [7]. A tracheostomy was performed on the 12th day of admission, and the patient's general condition and respiratory status gradually improved as he remained intubated. However, on the 24th day of admission, he suddenly developed ventilation failure and cyanosis, temporarily failing into respiratory and cardiac arrest and requiring cardiopulmonary resuscitation for 40 minutes, which restored his spontaneous circulation. While the cause of the sudden cardiac arrest remains unknown, the patient’s general condition, vital signs, and laboratory findings steadily improved, and mechanical ventilation was discontinued on the 34th day (Figure 2).

**Figure 3:**
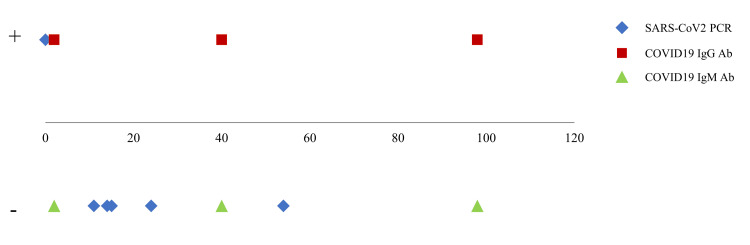
Qualitative findings of COVID-19 viral PCR, IgG, and IgM antibodies during the clinical course. Viruses were totally eliminated within two weeks (blue squares). The IgG antibody (red squares) was strongly positive on the day following admission, while the IgM antibody (green triangles) was negative, suggesting early seroconversion at the time of admission. PCR: polymerase chain reaction.

Supportive care and rehabilitation were continued as the patient's ability to perform physical activities improved, and the patient was discharged on the 53rd day. Virological measurements were obtained during treatment in the ICU and at follow-up outpatient clinic visits. Viral infection was diagnosed on the day of admission by PCR, and the same test was negative on the 11th day. In addition, the patient's blood analysis results on the second day revealed strong positivity for IgG antibodies against COVID-19, and this result persisted for over one year. However, the patient was negative for IgM antibodies throughout his clinical course. These antibody assays were performed using the Driven-Flow® COVID-19 IgG/IgM Antibody Test (Alfa Scientific Designs, Inc., Poway, CA) (Figure 3).

**Figure 4:**
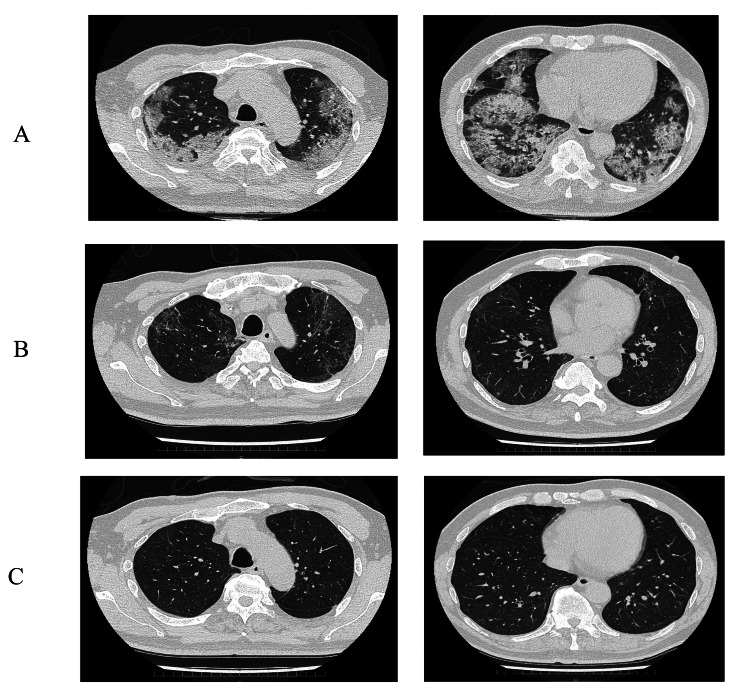
CT scans of the patient at admission (A) and at the one-month (B) and six-month (C) follow-up visits. These images show the time course of the patient's dissolving bilateral lung opacities.

Although the specific viral strain and its mutations may be associated with the clinical features, genomic analysis of the viral RNA was not performed. To screen for the potentially exacerbating factors of viral pneumonia as well as to rule out other causes of acute respiratory decompensation, radiological studies and peripheral blood autoantibody tests were performed during the treatment in the ICU as well as in the follow-up outpatient clinic. A CT scan showed gradual improvement in lung opacities, which were restored to almost normal after one year of follow-up (Figure 4).

Peripheral blood was drawn on the 36th day after admission and showed elevated serum levels of Sjögren's syndrome antigen A (SS-A) and Sjögren's syndrome antigen B (SS-B) autoantibodies (194.5 U/mL and 15.5 U/mL, respectively). Follow-up of the autoantibodies was performed to determine whether the findings were transient effects or clinically relevant phenomena; notably, the patient's blood levels of SS-A and SS-B continued to increase over the next 20 months (Table 3).

**Table 3:** Concentrations of the patient's SS-A and SS-B autoantibodies. anti-SS-A: anti-Sjögren's syndrome antigen A antibodies, anti-SS-B: anti-Sjögren's syndrome antigen B antibodies.

	At admission	12 months follow-up	20 months follow-up
Anti-SS-A	194.5 U/mL	521.3 U/mL	341.3 U/mL
Anti-SS-B	15.5 U/mL	41.4 U/mL	32.9 U/mL

**Figure 5:**
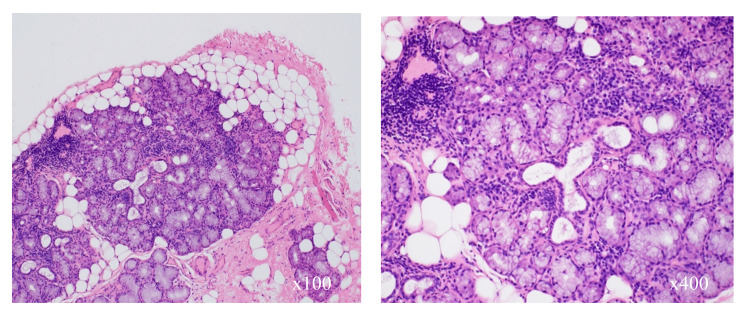
Salivary gland biopsy obtained during the follow-up of the patient. Magnified images with two foci in the area of 4 mm^2^ of salivary gland biopsy specimens showing infiltration of lymphocytes and plasma cells surrounding the conducting salivary glands. These findings are compatible with the histopathological diagnosis of Sjögren’s syndrome. Left: ×100; right: ×400.

Although the patient did not show any symptoms of eye discomfort or oral dryness, Schirmer's test result was positive. A salivary gland biopsy was performed to confirm the diagnosis of Sjögren’s syndrome. Histological samples from the patient’s lips showed the proliferation of plasma cells and lymphocytes in the area surrounding the conducting salivary glands, a finding compatible with the diagnosis of pathological chronic sialadenitis and Sjögren’s syndrome (Figure 5).

According to his medical record, there was no history of collagen vascular diseases. The patient is currently in a stable condition with no symptoms and is receiving treatment for pre-existing hypertension.

## Discussion

We successfully treated a patient with severe COVID-19 infection who required respiratory and circulatory support, and exhibited an elevated immune response despite early elimination of the virus. The patient was later diagnosed with Sjögren’s syndrome. The patient's early seroconversion of viral antibodies was suggestive of an elevated immune response to the viral infection, prompting us to screen for other antibodies. Because peripheral blood levels of SS-A and SS-B autoantibodies continued to rise, we performed a surgical biopsy of the patient’s salivary glands, leading to the histological confirmation of Sjögren’s syndrome. As viral infections are generally known to modify autoimmunity as well as an allergic response [8], and because COVID-19 itself can affect the autoimmune response in terms of recovery from a pathogenic insult [9], the severity of the disease at onset and the development of Sjögren’s syndrome soon after in this case may well be associated.

During the development of severe COVID-19, the innate immune system plays an important role in the release of inflammatory cytokines. Upon entry of the virus into the alveolar epithelium, transcriptional activation leads to the production of type I and III interferons [10], proinflammatory cytokines and chemokines, which exert diverse immunological effects on the lung tissue [11]. During the clinical course, this sequence can result in severe pneumonia and lead to acute respiratory decompensation. Furthermore, studies have suggested that immune complications may be a manifestation of COVID-19, and this is supported by the fact that therapeutic inhibition of IL-2 and tumor necrosis factor (TNF)-alpha may ameliorate hyperinflammation and that methylprednisolone treatment is associated with a more favorable outcome among COVID-19 patients who developed respiratory failure [12]. 

Pre-existing infections leading to the development of Sjögren’s syndrome have also been described previously [13]. Clinically, respiratory infections, including influenza A, paramyxoviridae, and rubella viruses, trigger autoimmunity through type II and IV hypersensitivity [14] that can lead to the development of Sjögren’s syndrome. Notably, although the patient discussed here did not have a medical history of collagen vascular disease, developed high serum levels of SS-A and SS-B autoantibodies that further increased during the follow-up period.

A major limitation of our study is that the patient's autoimmune status before developing COVID-19 is unknown; therefore, a link between the viral infection and the pathogenesis of Sjögren’s syndrome cannot be concluded. Studies also suggest that a large number of cases of Sjögren’s syndrome are left undiagnosed, with the prevalence of Sjögren’s syndrome in the general population being approximately 0.25% while only half of the patient population receives a clinical diagnosis [15]. However, our patient’s clinical presentation of severe COVID despite the early elimination of the virus and the absence of precipitating factors suggests an over-release of cytokines or previously unidentified immune responses as a trigger for these acute conditions and the identification of Sjögren’s syndrome. It should be stressed that although we did not obtain the genetic identity of the virus in this case, the D614G strain, which elicits higher rates of respiratory failure and mortality [16], was dominant at that time in our community [17].

With the continued occurrence of COVID-19 worldwide, there is increasing recognition of long-haul COVID. While some of these cases may be idiopathic [18], immune disorders triggered by viral infections warrant special attention.

## Conclusions

We report a case of severe COVID-19 that was followed by the development of Sjögren's syndrome, suggesting that hyperinflammation in the acute phase of infection might result from an enhanced immune response to viral infection. This also suggests that chronic responses in the post-infection period may trigger hyperinflammation and dysregulation of immunity, which can lead to the development of autoimmune diseases such as Sjögren's syndrome.
